# The food-borne pathogen *Campylobacter jejuni* depends on the AddAB DNA repair system to defend against bile in the intestinal environment

**DOI:** 10.1038/s41598-017-14646-9

**Published:** 2017-10-31

**Authors:** Christopher R. Gourley, Nicholas M. Negretti, Michael E. Konkel

**Affiliations:** School of Molecular Biosciences, College of Veterinary Medicine, Washington State University, Pullman, 99164-7520 WA USA

## Abstract

Accurate repair of DNA damage is crucial to ensure genome stability and cell survival of all organisms. Bile functions as a defensive barrier against intestinal colonization by pathogenic microbes. *Campylobacter jejuni*, a leading bacterial cause of foodborne illness, possess strategies to mitigate the toxic components of bile. We recently found that growth of *C. jejuni* in medium with deoxycholate, a component of bile, caused DNA damage consistent with the exposure to reactive oxygen species. We hypothesized that *C. jejuni* must repair DNA damage caused by reactive oxygen species to restore chromosomal integrity. Our efforts focused on determining the importance of the putative AddAB DNA repair proteins. A *C. jejuni addAB* mutant demonstrated enhanced sensitivity to deoxycholate and was impaired in DNA double strand break repair. Complementation of the *addAB* mutant restored resistance to deoxycholate, as well as function of the DNA double strand break repair system. The importance of these findings translated to the natural host, where the AddAB system was found to be required for efficient *C. jejuni* colonization of the chicken intestine. This research provides new insight into the molecular mechanism utilized by *C. jejuni*, and possibly other intestinal pathogens, to survive in the presence of bile.

## Introduction

Bile plays a critical role in the intestines of both humans and animals as a defensive barrier against colonization by pathogenic microbes. *Campylobacter jejuni*, which targets the intestines of susceptible hosts, is one of the leading bacterial causes of foodborne illness in the United States^1^. Numerous cases of *C. jejuni* illness result from the handling and consumption of foods cross-contaminated with raw poultry products. Indeed, the National Antimicrobial Resistance Monitoring System (NARMS) reported the presence of *C. jejuni* in 33% of retail chicken tested in 2014^2^. Alarmingly, 15% of the *C. jejuni* recovered from the poultry, as well as 27% of *C. jejuni* isolates recovered from humans, were resistant to the antibiotic ciprofloxacin. Given that poultry represent a natural reservoir for *C. jejuni*, research to expand our knowledge of *Campylobacter* colonization of chickens might prove helpful to the broiler industry in developing control methods that result in a decrease in the incidence of human campylobacteriosis. Moreover, studies are needed to further dissect the molecular mechanisms used by pathogens to overcome the toxic insult of intestinal bile.

Bile is produced in the liver and then drained into the intestine where it aids in digestion, absorption of fats, and excretion of waste products^3^. The primary bile acids (cholic acid and chenodeoxycholic acid) are synthesized in the liver from cholesterol in a multi-enzyme process. The secondary bile acids (lithocholic acid and deoxycholic acid) are produced as the result of metabolism of the primary bile acids by normal host intestinal bacteria. The average concentration of bile acids in the human intestine ranges from 0.2 to 2%^4^. The gastrointestinal tract of chickens is a natural reservoir of *C. jejuni* where the ceca serve as primary colonization sites. While the concentration of bile varies along the intestine of the chicken, bile concentration is greatest in the duodenum and upper jejunum^5^. Noteworthy is that bile secretion increases considerably in the duodenum of chickens during the first 21 days of life^6^.

Becoming increasingly evident is that *C. jejuni*, as a member of the Epsilonproteobacteria, does not possess certain mechanistic and regulatory pathways used by Gammaproteobacteria, including *Escherichia coli* and *Salmonella enterica* subspecies Typhimurium. In fact, predictions for the presence and function of *C. jejuni* DNA repair system components has largely been based on genomic comparisons looking for characterized homologues within closely related bacteria, including *Helicobacter pylori* ^7,8^. While *C. jejuni* clearly possess the gene encoding RecA, which has been demonstrated to be involved in repair of DNA double strand breaks using homologous recombination in many bacterial species, it does not harbor the *recBCD* genes involved in DNA repair in bacteria such as *E. coli*. Rather, researchers have postulated that *C. jejuni* encodes for two recombination repair proteins termed AddA and AddB^8,9^. Previous work with *Bacillus subtilis* has demonstrated that the AddA and AddB proteins function in place of the RecBCD complex to conduct the nuclease, helicase, and Chi recognition activity necessary for homologous recombination^10^. While it has been speculated that putative AddA and AddB homologues may function similarly in homologous recombination repair in *C. jejuni*, no experimental evidence of function or biological relevance has been described for these proteins in *C. jejuni*. Moreover, *Campylobacter* spp. lack the LexA repressor necessary to elicit a global regulatory response to DNA damage through the SOS response^11,12^. Therefore, beyond other Epsilonproteobacteria, cross species comparisons and predictions for response to DNA damage are difficult to make for *C. jejuni*. This lack of knowledge only reinforces the importance of more completely describing the mechanisms, regulation, and biological relevance of DNA repair in *Campylobacter* spp.

In a previous report, we found that the continuous growth of *C. jejuni* in medium with a physiological concentration of the bile salt deoxycholate induces the production of reactive oxygen species (ROS), which in turn, causes DNA damage (double strand breaks)^13^. Based on these findings, we hypothesized that *C. jejuni* must repair the ROS-induced DNA damage to promote survival. Given the paucity of detailed information regarding *C. jejuni* DNA repair systems, we decided to investigate the necessity of DNA repair in *C. jejuni*. Specifically, the purpose of this study was to determine if two putative DNA repair genes, designated CJH_07760 and CJH_07765 in *C. jejuni* strain F38011, function in DNA double strand break repair and play a role in the natural host (chickens). While RecA has been identified in *C. jejuni*, its partners in DNA homologous recombination have not been identified, and we suspected that the proteins encoded by the CJH_07760 and CJH_07765 genes are homologs of the AddA and AddB proteins demonstrated to work with RecA in other bacteria. In sum, this work revealed that the *Campylobacter* AddAB proteins contribute to DNA double strand break repair and are necessary for efficient colonization of chickens (the natural host).

## Results

### Description of *C. jejuni* AddA and AddB encoding genes

Given the absence of genes encoding RecBCD in *C. jejuni*, we focused our efforts on the *addA* and *addB* genes that are *recBCD* analogs^8^. A detailed comparison of the putative *C. jejuni addA* and *addB* genes and the products they encode are presented in Supplementary Table 1. The putative *addA* gene in the *C. jejuni* F38011, 81–176, and NCTC 11168 clinical strains is 2766 nucleotides in length and is predicted to encode a protein of 921 aa, while the putative *addB* gene is 2367 nucleotides in length and is predicted to encode a protein of 788 aa. The deduced aa sequence of the putative AddA protein from the *C. jejuni* F38011 strain exhibits approximately 48% similarity and 30% identity to the AddA protein from *H. pylori* strain 26695 and the deduced amino acid sequence of the putative AddB protein from the *C. jejuni* F38011 strain exhibits approximately 46% similarity and 29% identity to the AddB protein from *H. pylori* strain 26695. Based on these analyses, the putative *addA* and *addB* genes are highly conserved amongst *C. jejuni* isolates.

### The putative *addA* and *addB* genes are constitutively expressed in response to deoxycholate

Based on the analysis of RNA-Seq data and inspection of the distribution and number of reads corresponding to the putative *addA* and *addB* genes from *C. jejuni* strain F38011^13^, the *addAB* genes are likely to be contained in an operon where *addB* is upstream of *addA* (Supplementary Figure 2). Given that continuous growth of *C. jejuni* in deoxycholate results in DNA double strand breaks and the hypothesis that *C. jejuni* must repair the ROS-induced DNA damage to promote survival, quantitative reverse transcription PCR (RT-qPCR) was initially performed to determine if these two putative DNA repair genes are upregulated in response to growth in deoxycholate-supplemented medium over time. No change was observed in the expression levels of the putative *addA* and *addB* genes in *C. jejuni* strain F38011, whether in MH with 0.05% deoxycholate or MH alone (not shown). The levels of expression of the two genes were approximately 15 to 20-fold less than that for the oxidative stress response control (*rpoA*)^14^. Based on the result of the RT-qPCR analysis, it was concluded that the putative *addA and addB* genes are constitutively expressed in response to *C. jejuni* growth in MH broth and in MH broth supplemented with a moderate level (0.05%) of deoxycholate.

### The putative *addA* and *addB* genes from *C. jejuni* functionally complement an *E. coli ΔrecBCD* strain

**Figure 1:**
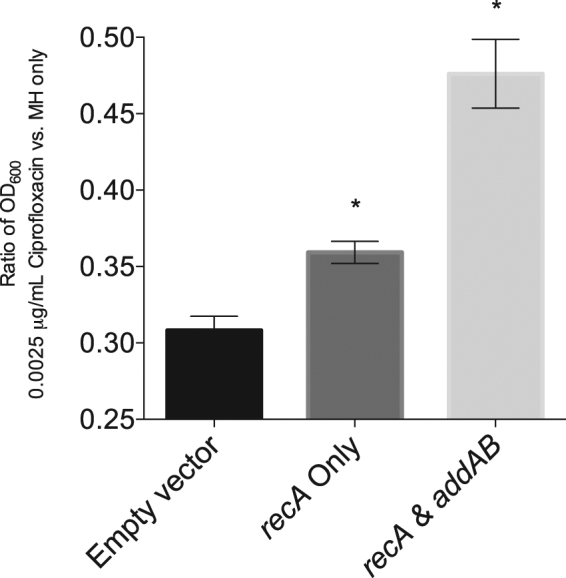
Addition of *C. jejuni recA* and putative *addAB* genes functionally complement an *E. coli recBCD* mutant. Given the high level of identity in the *addA* and *addB* gene sequences amongst the *C. jejuni* strains, we arbitrarily selected the putative *addA* and *addB* genes from *C. jejuni* strain 81–176 for these experiments. *E. coli* strain V3060 was transformed with an empty vector, with *recA* from *C. jejuni* strain 81–176, or with *addAB* and *recA* from *C. jejuni* strain 81–176. The cultures were grown in triplicate in LB broth for 18.5 hours with or without 0.0025 µg/mL of ciprofloxacin. Shown is the mean ratio (with ciprofloxacin/without ciprofloxacin) and standard deviation of the OD_600_ between the two culture treatments. A significant difference was observed in ciprofloxacin resistance in the *E. coli* strain harboring the *C. jejuni recA* gene alone and the *E. coli* transformed with the *C. jejuni recA* and *addAB* genes (*p < 0.05) compared to *E. coli* with the empty vector. An ANOVA followed by Tukey’s multiple comparisons test was done to evaluate significance.

The RecBCD enzyme of *E. coli* is essential for DNA double strand break repair and genetic recombination of linear DNA. The AddAB proteins are related to RecBCD, whereby the AddA protein essentially corresponds to RecB and the AddB protein to RecC^15^. To determine if the recombination DNA repair system from *C. jejuni* could functionally complement an *E. coli* strain with a *recBCD* deletion, a ciprofloxacin sensitivity assay was performed with a number of *E. coli* transformants. The *E. coli ΔrecBCD* mutant (strain V3060) was transformed with either an empty vector, a vector harboring only the *C. jejuni* putative *recA gene*, or a vector containing the *C. jejuni* putative *addA*, *addB*, and *recA* genes. All genes were driven by the T7 promoter. A ciprofloxacin sensitivity assay was then performed. Ciprofloxacin is a quinolone antibiotic that inhibits the function of DNA gyrase, eventually leading to an increase in DNA double strand breaks. Based on the literature and the fact that RecA and RecBCD are known to be involved in DNA double strand break repair, functional complementation of an *E. coli* RecBCD mutant was expected to demonstrate greater resistance to ciprofloxacin than the mutant transformed with an empty vector. Consistent with functional complementation, introduction of the putative *recA* gene into the *E. coli ΔrecBCD* mutant provided the isolate with increased ciprofloxacin resistance when compared to the *E. coli* mutant harboring the empty vector, but the *E. coli ΔrecBCD* isolate transformed with the *C. jejuni* putative *addA*, *addB*, and *recA* genes demonstrated even greater resistance to ciprofloxacin (Fig. 1). These findings demonstrate that the *C. jejuni* putative *addA* and *addB* genes functionally complemented the RecBCD enzyme of *E. coli*. Based on these findings, we designated CJH_07760 and its homologues in other *C. jejuni* strains as the gene encoding *C. jejuni* AddA and CJH_07765 and its homologues in other *C. jejuni* strains as the gene encoding AddB.

### The AddAB system is functional in *C. jejuni*

To determine if the AddAB system is functional in *C. jejuni*, gene deletions were generated in *addA*, *addB*, and both *addA* and *addB* in strain F38011. As mentioned previously, a mutation in *addB* is likely polar on *addA* since the two genes are contained in an operon, where *addB* is upstream of *addA*. The prRNA-*cysM*_prom_-*addAB*-hygromycin vector was also generated to test for AddAB functional complementation. This vector was designed to allow for wild-type copies of the *addAB* genes to be inserted into the chromosome whereby they are constitutively expressed from the *cysM* promoter. The prRNA-*cysM*_prom_-*addAB*-hygromycin construct was confirmed by restriction enzyme digests and sequencing. Similar to the assays performed earlier with the *E*. *coli* transformants, the *C. jejuni addA*, *addB*, and *addAB* deletion mutants were expected to demonstrate greater sensitivity to ciprofloxacin than the wild-type strain and complemented isolate, as treatment with this antibiotic results in DNA double strand breaks by inhibiting DNA gyrase. As predicted, the minimal inhibitory concentration (MIC) of ciprofloxacin for the *C. jejuni* F38011 wild-type strain was 0.125 μg/mL, whereas the MIC for the *addA* mutant, *addB* mutant, and *addAB* mutant was 0.03125 μg/mL. The MIC for the *C. jejuni addAB* mutant harboring the chromosomal copies of the *addAB* genes was identical to the wild-type strain (0.125 μg/mL). Collectively, these assays show that the AddAB proteins function to promote *C. jejuni* survival under conditions that generate DNA double strand breaks.

### Deoxycholate results in DNA double strand breaks in *C. jejuni*

**Figure 2:**
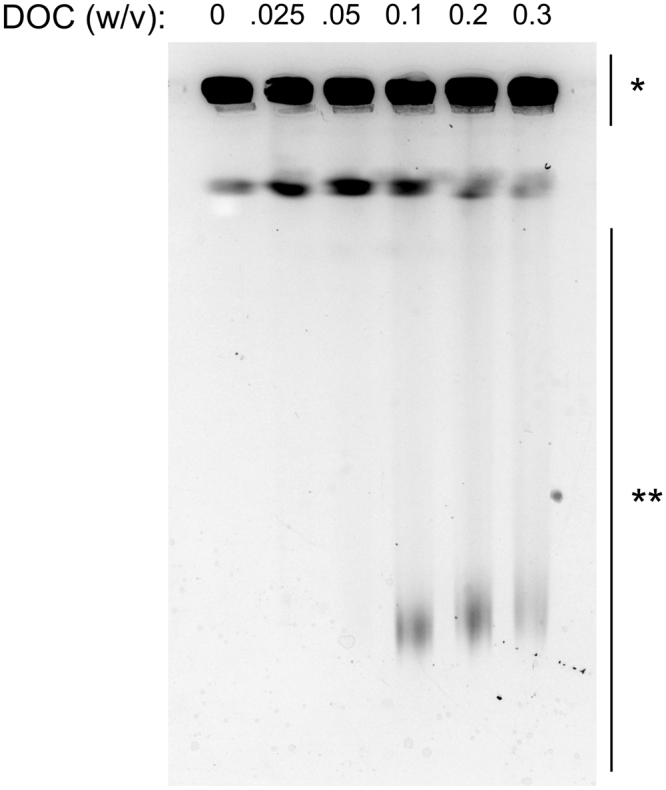
There is a threshold concentration of deoxycholate (DOC) needed to induce DNA damage. *C. jejuni* strain F38011 was grown for 20 hours in MH broth with 0.025% to 0.3% (w/v) DOC. Samples were collected and subjected to pulsed-field gel electrophoresis to evaluate DNA integrity. Limited to no damage was observed at a concentration less than or equal to 0.05% DOC, while damage was apparent in 0.1% DOC. One asterisk (*) indicates intact DNA, and two asterisks (**) indicate DNA fragments. Contrast is enhanced for clarity, see Supplementary Figure 3 for original image.

Although previous work in the laboratory had revealed that *C. jejuni* growth in medium supplemented with deoxycholate resulted in DNA double strand breaks, it was not clear the minimum dose required to generate the double strand breaks and whether the effect was dose-dependent. Briefly, *C. jejuni* were grown in MH broth alone as well as MH broth supplemented with different concentrations of deoxycholate, after which, pulsed-field gel electrophoresis (PFGE) was performed to detect DNA double strand breaks. The basis of this assay is that the fragmented DNA migrates in the agarose gel during PFGE, whereas the intact DNA does not migrate in the gel^16^. Fragmented DNA was readily visualized in samples prepared from *C. jejuni* grown in medium containing ≥0.1% deoxycholate (Fig. 2). In summary, *C. jejuni* growth in medium with 0.1% deoxycholate results in DNA double strand breaks that are readily detectable by PFGE.

### The AddAB system contributes to *C. jejuni* resistance to deoxycholate

**Figure 3:**
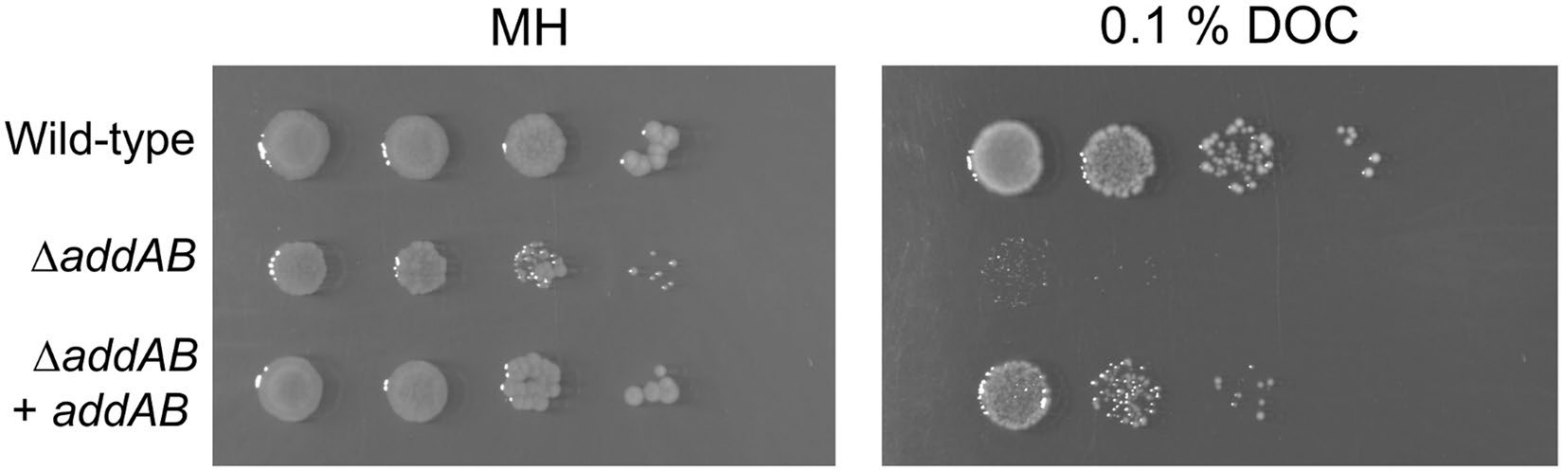
The *C. jejuni addAB* mutant demonstrates enhanced sensitivity to deoxycholate. Cultures of the *C. jejuni* F38011 wild-type strain, *addAB* mutant, and *addAB* mutant harboring *addAB* in *cis* (complemented isolate) were adjusted to an OD_540_ of 0.1. Ten-fold serial dilutions were spotted on to either MH agar or MH agar with 0.1% deoxycholate (DOC), and growth was evaluated after 48 hours of incubation.

Relevant to the colonization of a host, *C. jejuni* have the ability to resist bile. Given that the MIC of deoxycholate for *C. jejuni* was above the critical micelle concentration, the inhibitory concentration for the *C. jejuni* wild-type strain and *addAB* mutant to deoxycholate could not be determined. Regardless, the *C. jejuni* wild-type strain and *addAB* mutant were tested for sensitivity to 0.1% deoxycholate using a plate assay. Similar to the ciprofloxacin sensitivity assays, the *C. jejuni addAB* mutant demonstrated much greater sensitivity to deoxycholate versus the wild-type strain (Fig. 3). To confirm that the AddAB proteins are responsible for increased resistance to deoxycholate, the *C. jejuni addAB* mutant harboring the rRNA-*cysM*_prom_-*addAB*-hygromycin insertion was tested for functional complementation as judged by its sensitivity to deoxycholate. Consistent with the ciprofloxacin sensitivity assays, the *C. jejuni addAB* gene deletion mutant harboring a chromosomal copy of the wild-type *addAB* genes restored deoxycholate resistance (Fig. 3). Noteworthy is that the colonies formed by the *C. jejuni addAB* gene deletion mutant appeared smaller in size than the colonies formed by the *C. jejuni* wild-type strain and *addAB* mutant harboring *addAB* in *cis* (complemented isolate). However, additional assays revealed that the growth rates of the *C. jejuni* wild-type strain, *addAB* mutant, and *addAB* complemented isolate were indistinguishable from one another when grown in MH broth. Based on these data, we concluded that the *addAB* gene deletion confers deoxycholate sensitivity. Collectively, these data suggest that exposure to deoxycholate induces stress (*e.g*., DNA damage), and that the *C. jejuni* DNA homologous recombination repair system must respond to promote bacterial survival.

### The *C. jejuni* AddAB proteins are involved in DNA repair

**Figure 4:**
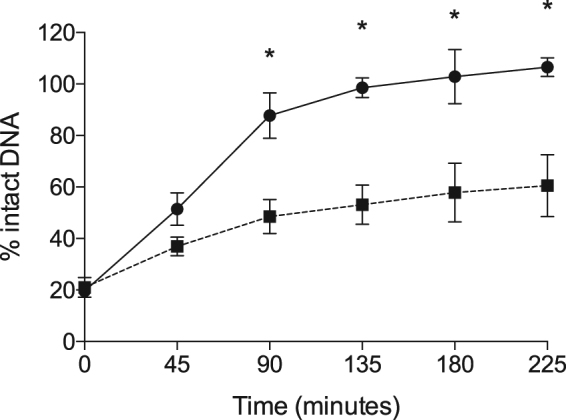
The *C. jejuni addAB* mutant is deficient in DNA double strand break repair. DNA double strand breaks were introduced by a 15-minute treatment with ciprofloxacin, after which the bacteria were transferred to MH broth. Aliquots were collected every 45 minutes to evaluate DNA double strand break repair by pulsed-field gel electrophoresis. A bacterial sample was collected prior to ciprofloxacin treatment as a negative control (untreated sample). Intact DNA was quantitated using relative band intensity, with the untreated sample set to 100%. Three biological replicates ± standard deviation are plotted for each time point. Significance between the *C. jejuni* wild-type strain (•) and the *addAB* mutant (▪) was calculated for each time point by one-way ANOVA followed by Sidak’s multiple comparisons test, and indicated by an asterisk (*p < 0.05).

To demonstrate that the AddAB system plays a role in DNA double strand break repair, *C. jejuni* were treated with ciprofloxacin for 15 minutes to introduce DNA double strand breaks, washed, and then incubated in MH broth to allow DNA repair. DNA repair in the *C. jejuni* wild-type strain versus *addAB* deletion mutant was then assessed by PFGE (Fig. 4). Evident from the gels was that the *C. jejuni addAB* deletion mutant was impaired in DNA double strand break repair. This finding supports the notion that the AddAB proteins could protect *C. jejuni* from the damaging effects caused by growth in deoxycholate, presumably by repairing DNA double strand breaks.

### The *C. jejuni* AddAB system is required for the efficient colonization of chickens

**Figure 5:**
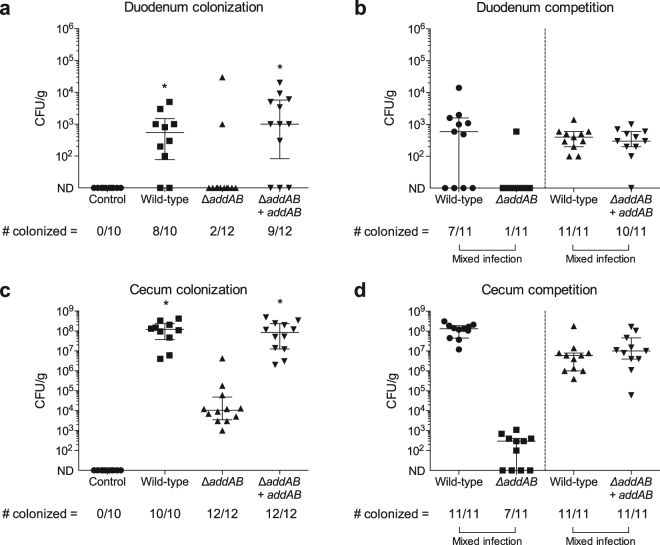
The AddAB DNA repair system of *C. jejuni* is essential for efficient colonization of chickens. The experiment was performed as outlined in ‘Materials and Methods.’ Panels (a) and (b): *C. jejuni* recovered from the duodenum, inoculated either alone (**a**) or in competition (**b**) with equal proportion of the *C. jejuni* wild-type strain and *addAB* mutant (*∆addAB*), or equal proportion of the *C. jejuni* wild-type strain and complemented *addAB* mutant (∆*addAB* + *addAB*). Panels (c) and (d): *C*. *jejuni* recovered from the cecum either inoculated alone (**c**) or in competition (**d**). ND indicates that no *C*. *jejuni* were detected (limit of detection > 10^2^ CFU/g). The horizontal bar with whiskers represents the median level of colonization and the interquartile range. The asterisk (*) indicates a significant difference (*p* < 0.05) in the CFU recovered versus the *C. jejuni addAB* mutant, as judged by a nonparametric Kruskal-Wallis test followed by Dunn’s test for multiple comparisons. *C. jejuni* was not recovered from any of the uninoculated chickens.

*Campylobacter* species frequently colonize the intestines of many wild and domestic birds. Based on the observation that the AddAB system confers deoxycholate resistance *in vitro*, experiments were performed to determine if the AddA and AddB proteins are necessary for *C. jejuni* survival in the chicken gut. Chickens were divided into 6 groups and inoculated with motile isolates of *C. jejuni* as outlined in ‘Materials and Methods.’ All chickens were euthanized at 14 days post-inoculation and the number of *C. jejuni* per gram of duodenal and cecal material was determined. Significant differences were observed in the level of colonization for a *C. jejuni* wild-type strain and the *addAB* mutant used in this study (Fig. 5). The *C. jejuni* wild-type strain was recovered from the duodenum of 8 of 10 birds, whereas the *addAB* mutant was recovered from the duodenum of only 2 of 12 birds (Fig. 5a). Similarly, the *C. jejuni* wild-type strain colonized the ceca of birds at a significantly higher level than the *addAB* mutant (Fig. 5c). Noteworthy is that the *C. jejuni addAB* mutant harboring the rRNA-*cysM*_prom_-*addAB*-hygromycin insertion was recovered from the duodenum of 9 of 12 birds and colonized the ceca of birds at a significantly higher level than the *addAB* mutant (Fig. 5a and c). Competition experiments showed that the *C. jejuni* wild-type strain and the *C. jejuni addAB* mutant harboring the rRNA-*cysM*_prom_-*addAB*-hygromycin insertion were equally recovered from both the duodenums and ceca of co-infected birds (Fig. 5b and d). Strikingly, the *C. jejuni* wild-type strain strongly outcompeted the *addAB* mutant in both duodenums and ceca of co-infected birds (Fig. 5b and d)*. C. jejuni* was not recovered from any of the mock inoculated chickens. Based on these data, we concluded that the AddAB proteins function to promote *C. jejuni* colonization/survival in chickens.

### *C. jejuni* infection of chickens does not alter the bile concentration in the intestine

In parallel with the *in vivo* experiments to determine the importance of the AddAB DNA repair system in *C. jejuni* colonization of chickens, assays were performed to determine if *C. jejuni* infection alters bile concentration in the intestine. Known is that the concentration of bile varies along the intestine of the chicken, and that bile salt concentration is greatest in the duodenum and upper jejunum^5^. The release of bile into the duodenum is stimulated by the sensing of acidic pH, fatty acids, and bile salts by cells of the duodenum that subsequently release stimulating hormones, such as cholecystokinin, that triggers gallbladder contraction^3^. Therefore, bile concentration in the duodenum of an individual bird fluctuates with the presence or absence of digesta.

**Figure 6:**
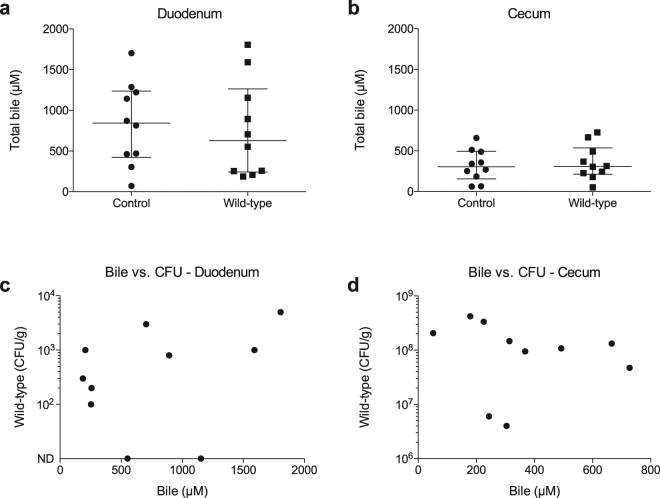
Infection of chickens does not alter the bile concentration in the intestine. Total bile concentration was determined in the duodenum (Panel a) and cecum (Panel b) of mock inoculated chickens (control) and chickens inoculated with *C. jejuni* wild-type strain using a bile acid assay kit (Sigma-Aldrich). The total CFU recovered from *C. jejuni* wild-type inoculated chickens was compared to the concentration of bile in the duodenum (Panel c) and the cecum (Panel d). The concentration of bile was independent from the number of live *C. jejuni* recovered. The horizontal bar with whiskers represents the median bile concentration and the interquartile range.

The concentration of bile was determined in the *C. jejuni*-inoculated chickens and mock inoculated chickens from samples collected when the colonization experiment was terminated (samples were collected at the time of necropsy). While the amount of total bile was observed to vary amongst the birds, a greater level of bile was observed in the duodenums than in the ceca (Fig. 6a and b). Analysis of the 20 samples (collected from the mock inoculated and *C. jejuni*-inoculated chickens) revealed that the bile concentration ranged from 70.6 µM to 1805.5 µM in the duodenums. Noteworthy is that ~1206 µM of deoxycholate (0.05%) was used in the *in vitro* assays in this study. *C. jejuni* colonization of the chickens had no effect on total bile concentration in either the duodenums or ceca (Fig. 6a and b). In addition, no correlation was observed between *C. jejuni* CFU and bile concentration in either the duodenums or ceca (Fig. 6c and d). Collectively, these findings suggest that the *C. jejuni* colonization of chickens does not alter intestinal bile concentration and, *vice versa*, the concentration of bile does not alter *C. jejuni* colonization of the duodenum or ceca (that is, the total number of recoverable CFU).

## Discussion

Chickens are a natural host for *Campylobacter* species, and *C. jejuni*, in particular, colonizes the avian gastrointestinal tract of chicks by two to four weeks of age^17^. Based on the efficiency of *C. jejuni* chicken colonization (*i.e*., as few as 35 CFU is sufficient for the colonization of chicks), it is evident that certain strains possess strategies that promote efficient colonization and survival within this host. Over the past two decades, researchers have expended a significant amount of effort to identify and characterize proteins that facilitate *C. jejuni* colonization and survival in the chicken gut. While a variety of *C. jejuni* proteins have been identified that contribute to these processes, including proteins that are involved in motility, cell adherence, modification of bacterial cell surface structures, etc., less attention has been paid to the proteins involved in enhancing cellular survival. As such, the discovery of a previously unrecognized survival response mechanism to bile salts (*e.g*., maintenance and repair of the cellular DNA) could represent a strategy utilized by other pathogens to maintain homeostasis in the gut. Efficient and accurate repair of DNA damage is crucial to ensure genome stability and cell survival.

This work was based on the original finding that deoxycholate triggers production of reactive oxygen species (ROS) and causes DNA damage^18,19^, including the DNA double strand breaks shown in this study. Based on the absence of any well-characterized DNA repair system in *C. jejuni*, and the fact that the repair of DNA double strand breaks predominantly involves homologous recombination, our efforts were focused on the characterization of the putative AddAB system. We first demonstrated that the *C. jejuni addA* and *addB* genes encode proteins that are functionally analogous to the RecBCD proteins of *E. coli* using well-accepted laboratory assays. More specifically, an *E. coli recBCD* mutant harboring *C. jejuni addAB* and *recA* was found to demonstrate greater resistance to ciprofloxacin than the *E. coli* mutant harboring the empty vector. The utilization of *C. jejuni* AddAB proteins in *E. coli* strongly supports the assertion that these genes function as part of the homologous DNA repair system in *C. jejuni*. Next, the *C. jejuni addAB* mutant was found to be more sensitive to both ciprofloxacin and deoxycholate treatment than the wild-type strain. Importantly, the *C. jejuni addAB* mutant transformed with the *addAB* genes was successfully complemented *in vitro*, as judged by the ciprofloxacin and deoxycholate sensitivity assays. The importance of the AddAB proteins were then demonstrated *in vivo*, as the *C. jejuni addAB* mutant was impaired in gut colonization when compared to the wild-type strain. Moreover, the *C. jejuni addAB* mutant harboring the rRNA-*cysM*_prom_-*addAB*-hygromycin insertion colonized the duodenum and cecum at a level that was nearly indistinguishable from that of the wild-type strain. Collectively, the data demonstrate that the AddAB system is critical for *C. jejuni* to colonize chickens.

The magnitude of the effect of *addAB* gene deletion on *C. jejuni* colonization is a vital observation for several reasons. First, organisms have evolved overlapping mechanisms for homologous recombination repair, and this redundancy in repair mechanisms highlights the potentially devastating impact on fitness posed by unrepaired lesions. More specifically, bacteria frequently possess two lesion dependent pathways to enable RecA loading onto single stranded DNA for homologous recombination repair. The Rec(F)OR pathway is utilized to repair DNA gaps (single strand breaks) and the AddAB (or RecBCD) pathway is utilized in the presence of DNA double strand breaks^20,21^. Based on sequence similarity to *H. pylori* genes, *C. jejuni* is believed to encode RecO and RecR homologues^7,22^, so it is possible that recombination repair involving these proteins may occur in *C. jejuni*. However, it must also be noted that DNA single strand breaks can progress to double strand breaks if not properly repaired^20^. Accordingly, our work clearly demonstrates the critical importance of the *C. jejuni* AddA and AddB double strand break repair proteins. Second, due to its critical role in the natural host, one might expect the *addAB* genes to be upregulated in response to the stressor that is instigating the DNA double strand breaks. This is the case in *Coxiella burnetii*, an obligate intracellular pathogen, that upregulates *addAB* homologues in response to macrophage induced oxidative stress^23^. However, work on *Bacillus subtilis* has revealed that *addAB* gene expression is independent of DNA-damaging agents^24^. Although the *addAB* genes were found not to be upregulated in *C. jejuni* in response to growth in deoxycholate from 10 to 16 hours, it is possible that these genes are upregulated at other times or in response to other environmental conditions. In addition to DNA breaks, deoxycholate-mediated oxidative damage likely causes other types of DNA damage. In agreement with this proposal, the *uvrC* gene is upregulated in *C. jejuni* grown in medium containing deoxycholate^13^ and ovine bile^25^. UvrC is an excision nuclease protein and part of the UvrABC complex involved with nucleotide excision repair^26^. Recent work indicates that this pathway, in addition to the base excision repair pathway, defends against oxidative damage^27,28^. Also worth noting is that *C. jejuni* strains demonstrate different levels of sensitivity to deoxycholate, and based on this observation, we hypothesize that an isolate with greater resistance to deoxycholate would have a selective advantage for establishing colonization and potentially causing disease. Although no correlation was observed between *C. jejuni* CFU and bile concentration in the duodenums of birds, curious is that the ceca, where the concentration of bile is low, are the preferential gastrointestinal colonization sites for this organism (see Fig. 5). Finally, given the critical role of the AddAB system in *C. jejuni* in poultry, we hypothesize that the AddAB proteins play a role in early human infection, where the pathogen survives and multiplies in the presence of bile in the upper region of the small intestine^29^.

Researchers have spent significant time in defining *C. jejuni* colonization factors, because poultry products (or the chicken reservoir as a whole) are estimated to be responsible for up to 80% of human campylobacteriosis cases^17^. However, researchers have also observed that *C. jejuni* isolates have different colonization potentials. In brief, some isolates appear unable to colonize 14-day old chickens, whereas others colonize chickens but are eliminated from the gut, and yet others show efficient and sustained colonization of chickens. While the ability of *C. jejuni* to colonize birds is without a doubt multifactorial, many of the factors that contribute to colonization have likely not been identified. We initiated this study with the *C. jejuni* F38011 and 81–176 clinical strains. However, in our preliminary work, we observed that *C. jejuni* strain 81–176 poorly colonized chickens (n = 3 experiments, with a mean colonization rate = 3.06 × 10^3^ CFU per gram of cecal content at 10 days post-inoculation). At nearly the same time, we also noted that the *C. jejuni* 81–176 strain is more sensitive to deoxycholate than strain F38011. Noteworthy is that *C. jejuni* strain 81–176 was responsible for a point-source outbreak of campylobacteriosis associated with consumption of raw milk^30^, whereas the reservoir of *C. jejuni* strain F38011 is not known. The enhanced sensitivity of *C. jejuni* strain 81–176 to deoxycholate raises the possibility that the ability of an isolate to withstand and adapt to bile components may contribute to the variability in colonization capacity (or to its ability to establish a particular niche).

Pathogenic microbes that colonize the gut must cope with a wide-range of host defense mechanisms. Bile provides the host gut a formidable barrier to colonizing pathogens. Indeed, bile interacts with the cellular membranes of both mammalian and bacterial cells, causing membrane perturbation and oxidative stress. Previously, we found that the growth of *C. jejuni* in medium containing the bile salt deoxycholate resulted in elevated levels of intracellular ROS, which in turn, caused significant DNA damage^13^. To overcome this insult, *C. jejuni* adapt to the intestinal milieu (exposure to bile) through use of the RecA/AddAB DNA repair system. The importance of the DNA damage repair pathways *in vivo* is likely the basis for why mutations (gene deletions) in these systems have been utilized in a number of enteric bacteria for the generation of live, attenuated vaccine strains^31,32^. Regardless, based on our data, we conclude that functional DNA repair systems are necessary for organisms to maintain their chromosomal integrity. In other words, without repair of the chromosomal lesions introduced by the chemical insults found in the intestinal environment, *C. jejuni* would fail to replicate and transmission to a new host would cease.

## Materials and Methods

### Bacterial strains, vectors, and oligonucleotides

The bacterial strains, vectors, and oligonucleotides used is this study are listed in Table 1. *C. jejuni* were routinely cultured on Mueller-Hinton (MH) agar (Difco Brand, BD Biosciences, Sparks, MD) containing 5% citrated bovine blood (MHB agar) or in MH broth with orbital shaking under microaerobic conditions (5% O_2_, 10% CO_2_, 85% N_2_) at 37 °C. *E. coli* strains [Stellar^TM^ (Takara Bio, USA) and V3060] were routinely cultured on LB-Miller agar (Thermo Fisher Scientific, Waltham, MA) or in LB-Miller broth under aerobic conditions at 37 °C.

**Table 1:** Bacterial strains, vectors, and oligonucleotides used in this study.

Strain	
*C. jejuni* 81–176	Outbreak associated with raw milk^30^
*C. jejuni* F38011	Human case of diarrhea^37^
*E. coli* Stellar™	Takara (Clontech)
*E. coli* V3060	8
**Vector**	
pBSK-Kan2	38
pBSK-*addA* KO	This work
pBSK-*addB* KO	This work
pBSK-*addAB* KO	This work
prRNA-Hygro-*cysM* ^Pro^-*addAB*	This work^39^
pacycDUET^TM^−1	Novagen
pacycDUET-*recA*	This work
pacycDUET-*addAB* & *recA*	This work
Oligo name	Sequence
Mutant Generation Primers	
*addA* DWN T	5′-TATAGGGCGAATTGGGTACCCCTTCTTTAGCAATTTGCGCAG-3′
*addA* DWN B	5′-GATCGGATCCGGAAAATGAAATTCAAATTTTGGAAATATAAAC-3′
*addA* CAT T	5′-TTCATTTTCCGGATCCGATCTGCGCCCTTTAGT-3′
*addA* CAT B	5′-ATTAAAATGACTGCAGGTGTTCCTTTCCAAGTTAATTGCG-3′
*addA* UP T	5′-ACACCTGCAGTCATTTTAATTCCTTTTTATAAATGAGTTTATAAGG-3′
*addA* UP B	5′-GGGAACAAAAGCTGGAGCTCAAATTTCACAACTTTCCCTTAGTG-3′
*addB* DWN T	5′-TATAGGGCGAATTGGGTACCCTCTCTTACATCAAGCCTC-3′
*addB* DWN B	5′-GATCGGATCCGATGAAAATGCAAGCGTGG-3′
*addB* CAT T	5′-CATTTTCATCGGATCCGATCTGCGCCCTTTAGT-3′
*addB* CAT B	5′-TGCTCAATGACTGCAGGTGTTCCTTTCCAAGTTAATTG-3′
*addB* UP T	5′-ACACCTGCAGTCATTGAGCATTTAATTCGTAGC-3′
*addB* UP B	5′-GGGAACAAAAGCTGGAGCTCTGGCCTTATGGTATTTTGC-3′
Complementation Primers	
cysMprom XbaI FW	5′-ATATATTCTAGACATCAGTTTTATTGGTTTTGGTACTTTTTCAACTC-3′
cysMprom SphI BamHI RV	5′-ATAGGATCCGCATGCAATTTTAATATCCTTTTTTGTTTAATAATGATAGTTTTATAAAAG-3′
addBA F38011 sphI FW	5′-ATATATGCATGCATGAAATTAAGAATTTTTAGTTCTTCAAGACAAATTAGAG-3′
addBA_F38011 BamHI RV	5′-ATATATGGATCCTTATATTTCCAAAATTTGAATTTCATTTTCCAAACAATAAAC-3′
HygR EcoRI FW	5′-ATATATGCATGCATGAAATTAAGAATTTTTAGTTCTTCAAGACAAATTAGAG-3′
HygR PstI RV	5′-ATATATCTGCAGTTATCATGCCTTTCTTTGTCTGTATTC-3′
Cipro Sensitivity primers	
BamHI *addB* T	5′-TATATAGGATCCATGAAATTAAGAATTTTTAGCTCTTCAAGG-3′
SacI *addA* B	5′-TATATAGAGCTCTTATATTTCCAAAATTTGAATTTCATTTTCC-3′
NcoI *addB* T	5′-TATATACCATGGATGAAATTAAGAATTTTTAGCTCTTCAAGG-3′
PstI *addA* B	5′-TATATACTGCAGTTATATTTCCAAAATTTGAATTTCATTTTCC-3′
NdeI *recA* T	5′-TATATACATATGGATGATAATAAAAGAAAATCTCTAGAC-3′
KpnI *recA* B	5′-TATATAGGTACCTTATTCTTCTCCTTCGTCATCTTC-3′
RT-qPCR Primers	
RpoA RT-F	5′-CGAGCTTGCTTTGATGAGTG-3′
RpoA RT-R	5′-AGTTCCCACAGGAAAACCTA-3′
AddA_RT1F	5′-GGGTTTGGAATTTGATCATGTG-3′
AddA_RT1R	5′-ATGGAGTTGCCAACCTTGATT-3′
AddB_RT1F	5′-CCTTTCGAGTTTCCGCTTTC-3′
AddB_RT1R	5′-CGCTTAAAACCCTAGGTTCTGC-3′

### RNA extraction and quantitative reverse transcription PCR

*C. jejuni* strain F38011 was inoculated at an OD_540_ of 0.05 in 50 mL of MH broth and MH broth supplemented with 0.05% (w/v) sodium deoxycholate (Sigma-Aldrich, St. Louis, MO) in 125 mL flasks and incubated at 37 °C under microaerobic conditions with orbital shaking (220 rpm). *C. jejuni* was grown in MH and MH deoxycholate side-by-side in the same chamber and an aliquot of each culture was taken at 10, 12, 14, and 16 hours post inoculation. These time points were chosen because *C. jejuni* growth in medium with deoxycholate was inhibited after 12 hours compared with MH broth without deoxycholate (not shown). Bacteria from each aliquot were isolated by centrifugation, culture supernatants were removed, and pellets were snap frozen in liquid nitrogen. To ensure reproducible results, the time-course experiment was repeated twice. Total bacterial RNA was isolated using the Ambion Ribopure Bacteria kit and treated twice with the supplied DNAse (Thermo Fisher Scientific, Waltham, MA). All RNAs were stored in Elution Solution. First strand cDNA synthesis was conducted using the Invitrogen SuperScript™ III First-Strand Synthesis System for RT-PCR (Thermo Fisher Scientific, Waltham, MA) according to the manufacturer’s instructions. A total of 450–500 nanograms of RNA per sample was used as template, and separate reactions were conducted with and without reverse transcriptase for each sample. Applied Biosystems^TM^ *Power* SYBR™ Green PCR Master Mix (Thermo Fisher Scientific, Waltham, MA) was used according to the manufacturer’s instructions for RT-PCR reactions. Amplification was conducted using an Applied Biosystems^TM^ 7500 Fast Real-Time PCR System (Thermo Fisher Scientific, Waltham, MA) and data was analyzed using version 2.3 of the associated software. Reaction conditions were 95 °C for 10 min followed by 40 cycles of 95 °C for 1 minute and 60 °C for 1 minute. The RT-qPCR primers are listed in Table 1.

### Sensitivity of an *E. coli* Δ*recBCD* strain to ciprofloxacin when complemented with *C. jejuni addAB*

The *addAB* genes and *recA* gene were amplified from *C. jejuni* strain 81–176 (see Cipro Sensitivity primers, Table 1). Fragments were incorporated into the pacycDuet-1 vector to make two distinct vectors: pacycDuet-*recA* and pacycDuet-*addAB*-*recA*. *E. coli* V3060^8,9^ (Δ*recBCD*) was transformed by electroporation with pacycDuet-1, pacycDuet-*recA*, or pacycDuet-*addAB*-*recA*. Ciprofloxacin sensitivity was determined by inoculation of 5 mL of LB-Miller broth or 5 mL of LB-Miller broth with 0.0025 μg/mL ciprofloxacin-HCl (Alfa Aesar, Haverhill, MA). The cultures were grown for 18.5 hours with shaking at 37 °C, and then the OD_600_ was measured. Significant differences were determined by one-way ANOVA with Dunnett’s multiple comparisons test of culture OD_600_ ratios (with ciprofloxacin/without ciprofloxacin).

### Generation of *C. jejuni addA* and *addB* gene mutants and a complemented isolate

*C. jejuni addA*, *addB*, and *addAB* mutants were generated in strain F38011 using standard molecular biology techniques. The suicide vectors were constructed using In-Fusion Cloning (Takara Bio, USA) to link DNA regions flanking the gene of interest, a chloramphenicol cassette, and the pBSK-Kan2 vector. The flanking regions for the *addA* suicide vector were amplified by PCR from *C. jejuni* strain F38011, and the flanking regions for the *addB* and *addAB* suicide vectors were amplified from *C. jejuni* strain 81–176 (see Mutant Generation Primers, Table 1).

The complementation of the *C. jejuni* F38011 *addAB* mutant was achieved using a procedure similar to that described by Karlyshev and Wren^33^. Briefly, the *cysM* promoter (301 bp) and *addAB* genes (5129 bp) were amplified from *C. jejuni* strain F38011 and inserted between the rRNA flanking regions of prRNA-Hygro (the order is: rRNA upstream - XbaI - *cysM* promoter - SphI - *addAB* - BamHI - FLAG tag - EcoRI - Hyg^R^ Cassette - PstI - rRNA downstream) (Table 1, Complementation Primers, Supplementary Figure 1).

The vectors (for both gene deletion or complementation) were electroporated into the *C. jejuni* F38011 strain and *addAB* mutant, and transformed isolates were selected on MH blood agar with appropriate antibiotic. All electorportations were conducted using a Bio-Rad *E. coli* Pulser and 2 mm gap cuvettes at the 2.5 kV voltage setting. The mutant and complemented isolates were confirmed by PCR.

### *C. jejuni* ciprofloxacin sensitivity assays

The minimum inhibitory concentration (MIC) of ciprofloxacin was determined by inoculating 5 mL of MH broth containing a 2-fold serial dilution (from 0.5 μg/mL to 0.00390625 μg/mL) of ciprofloxacin with the *C. jejuni* F38011 wild-type strain, *addA* mutant*, addB* mutant*, addAB* mutant, and *addAB* mutant harboring the prRNA-*cysM*_prom_-*addAB*-hygromycin insertion. Cultures were grown with orbital shaking under microaerobic conditions (5% O_2_, 10% CO_2_, 85% N_2_) at 37 °C for 18 hours. The MIC was defined as the lowest concentration of ciprofloxacin that impaired growth (less culture density) compared to the MH only (no ciprofloxacin) control.

### *C. jejuni* deoxycholate sensitivity assays

*C. jejuni* was grown overnight in MH broth, pelleted, adjusted to an OD_540_ of 0.1 in PBS. Ten-fold serial dilutions were then performed in PBS. Four µL drops were spotted on to MH agar plates and MH agar plates containing 0.1% (w/v) sodium deoxycholate. Growth on the plates was evaluated after 48 hours of incubation.

### Pulsed-field gel electrophoresis and DNA-repair assays

DNA damage in response to deoxycholate was evaluated by growing *C. jejuni* strain F38011 in MH broth and MH broth with the indicated concentrations of sodium deoxycholate for 20 hours. Samples were collected and embedded in agarose plugs.

Ciprofloxacin inhibits the function of DNA gyrase and increases the likelihood of DNA double strand breaks. The ability to repair ciprofloxacin-induced damage was evaluated by inoculating an overnight culture of the *C. jejuni* F38011 wild-type strain and *addAB* mutant in MH broth at an OD_540_ of 0.4. A sample was collected immediately after dilution as a negative control sample. Ciprofloxacin was added at a concentration of 1 µg/mL and the cultures were incubated with orbital shaking under microaerobic conditions at 37 °C for 15 minutes. Cultures were pelleted at 10,000 x *g* for 10 minutes, washed in MH broth, pelleted again, and then suspended in fresh MH broth without ciprofloxacin. A sample was taken immediately after culture resuspension as the zero time point. Cultures were incubated with orbital shaking under microaerobic conditions at 37 °C and samples were taken every 45 minutes until 225 minutes post ciprofloxacin treatment. All samples were embedded in agarose plugs and prepared for Pulsed-Field Gel Electrophoresis (PFGE). PFGE was performed using a CHEF*-*DR^*®*^ III variable angle system (Bio-Rad, Hercules, CA), as outlined previously^34^. Typical run parameters consisted of a reorientation angle of 120° with a constant voltage of 120 V at 14 °C. The pulse times were ramped from 6.8 to 35.4 s over 19 hours. Gels were stained for 30 minutes in 3 µg/mL ethidium bromide and destained in water. Images were captured using a GE Healthcare ImageQuant LAS 4000 Mini system and processed using ImageJ (version 2.0.0-rc34).

### Leghorn chickens

Two-day old straight run Pearl-White Leghorn chickens (catalogue number PEAS) were obtained from Murray McMurray Hatchery (Webster City, IA). Chickens were housed in PLAS labs isolator units and provided food (Purina Flock Raiser Crumbles) and water *ad libitum*. Water was fortified with Quik Chik (Murray McMurray Hatchery) vitamin and electrolyte supplement for one week after receipt. All efforts were made to raise the animals humanely.

### *C. jejuni* colonization of chickens

Motility assays were performed as outlined elsewhere^35^. The chickens were divided into 6 treatment groups of 10–12 broiler chickens, with each group housed in a separate isolator unit. The treatment groups consisted of: 1) one negative control group (mock inoculated); 2) one positive control group inoculated with a wild-type strain of *C. jejuni*, strain F38011 (*C. jejuni*-inoculated group); 3) one group inoculated with the *addAB* mutant; 4) one group inoculated with the *addAB* mutant harboring the prRNA-*cysM*_prom_-*addAB*-hygromycin insertion; 5) an equal mix of the *C. jejuni* wild-type strain and *addAB* mutant; and 6) an equal mix of the *C. jejuni* wild-type strain and *addAB* mutant harboring the prRNA-*cysM*_prom_-*addAB*-hygromycin insertion. At 12 days of age, the chickens were orally inoculated with 1–2 × 10^7^ *C. jejuni* in 0.2 mL of MH broth or were mock inoculated with 0.2 mL of MH broth without bacteria. The chickens were housed for an additional 14 days after inoculation and then euthanized by carbon dioxide asphyxiation followed by cervical dislocation. A 1–2 inch section of the duodenum and one cecum were dissected from each bird. Each section of the duodenum and cecum was individually weighed, stomached in an equal volume of MH broth (1 mL broth per gram of content), and then serially diluted for enumeration on Campy Cefex Agar^36^. The samples from groups five and six were also plated on Campy Cefex Agar with 8 μg/mL chloramphenicol to enable determination of the colonization of the *addAB* mutant and *addAB* mutant harboring the prRNA-*cysM*_prom_-*addAB*-hygromycin insertion. Chicken experiments were performed in accordance with the National Research Council’s *Guide for the Care and Use of Laboratory Animals* (8th edition) and approved by the Washington State University Institutional Animal Care and Use Committee under protocol ASAF 4847.

### Measurement of total bile contents in the chicken intestine

Bile acid levels in the intestines of negative (uninoculated) and positive (inoculated with a wild-type *C. jejuni* strain) control groups were measured using the Bile Acid Assay Kit as outlined by the manufacturer (Sigma-Aldrich, St. Louis, MO). Fluorescence intensity (λ_ex_ = 530 nm/λ_em_ = 595 nm) was read using a VICTOR ×5 reader (Perkin-Elmer, Waltham, MA).

## Electronic supplementary material

Supplementary Figures
