# Supplementary material for: The food-borne pathogen Campylobacter jejuni depends on the AddAB DNA repair system to defend against bile in the intestinal environment
Source: Sci Rep. 2017 Oct 31;7:14777. doi: 10.1038/s41598-017-14646-9 (PMC5665897; doi:10.1038/s41598-017-14646-9)
Supplement: Supplementary file 1 — Supplementary Figures [file 41598_2017_14646_MOESM1_ESM.pdf]

The food-borne pathogen *Campylobacter jejuni* depends on the AddAB DNA repair system to defend against bile in the intestinal environment

Christopher R. Gourley[1], Nicholas M. Negretti[1], and Michael E. Konkel*

[1]These authors contributed equally to this work.

School of Molecular Biosciences, College of Veterinary Medicine, Washington State University, Pullman, WA, USA, 99164-7520.

*Correspondence and requests for materials should be addressed to: Dr. Michael E. Konkel, Biotechnology Life Sciences Building, Room 447, Washington State University, Pullman WA 99164, (+1) 509 335 5039; Fax (+1) 509 335 4159; Email: konkel@vetmed.wsu.edu

SUPPLEMENTARY FIGURES

Supplementary Figure 1. Construction of the prRNA-Hygro suicide vector used in this study. Briefly, regions of the rRNA gene cluster (800 bp upstream and 1093 bp downstream of the targeted insertion site) were PCR amplified and cloned into the pBSK-Kan2 vector. The 800 bp rRNA upstream region (rRNA Up) corresponds to nucleotides 39844 to 40643 and the 1093 bp rRNA downstream region (rRNA Down) corresponds to nucleotides 40650 to 41742 of *C. jejuni* strain F38011 (Accession CP006851, Version CP006851.1). The hygromycin resistance cassette was amplified from a synthetic DNA template (gBlock Gene Fragment from Integrated DNA Technologies, Coralville, Iowa) using sequence from Nirdnoy *et al*. (Nirdnoy et al., 2005). FLAG, FLAG epitope; ori, ColE1 origin of replication; KanR, Kanamycin resistance cassette; p cysM, *cysM* promoter; addAB, *addAB* gene sequences. Restriction enzyme sites as indicated (KpnI, XbaI, SphI, EcoRI, BamHI, PstI, and SacI).

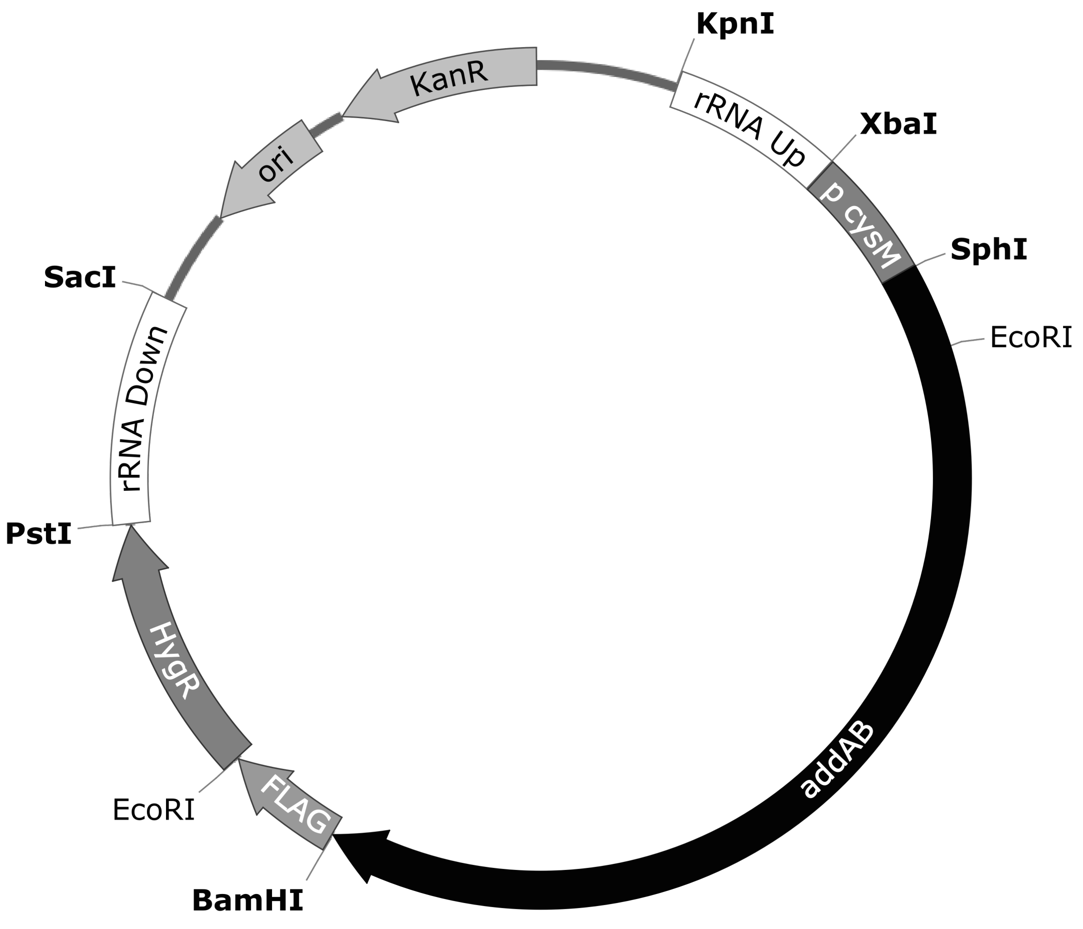

Supplementary Figure 2. The *C. jejuni addA* and *addB* genes are in an operon. All of the collected RNA-Seq reads for *C. jejuni* strain F38011 were compiled and mapped to the *C. jejuni* strain F38011 genome and visualized with GenomeView. *addAB* has a much lower expression level compared to the flanking genes. In the graphic below, only 50 reads were stacked for clarity, and there are additional non-visible reads for the *rplM* and CJH_07770 genes (red line). No reads were detected that overlapped between *addA* and *rplM*, while we detected many overlapping reads between *addA* and *addB*.

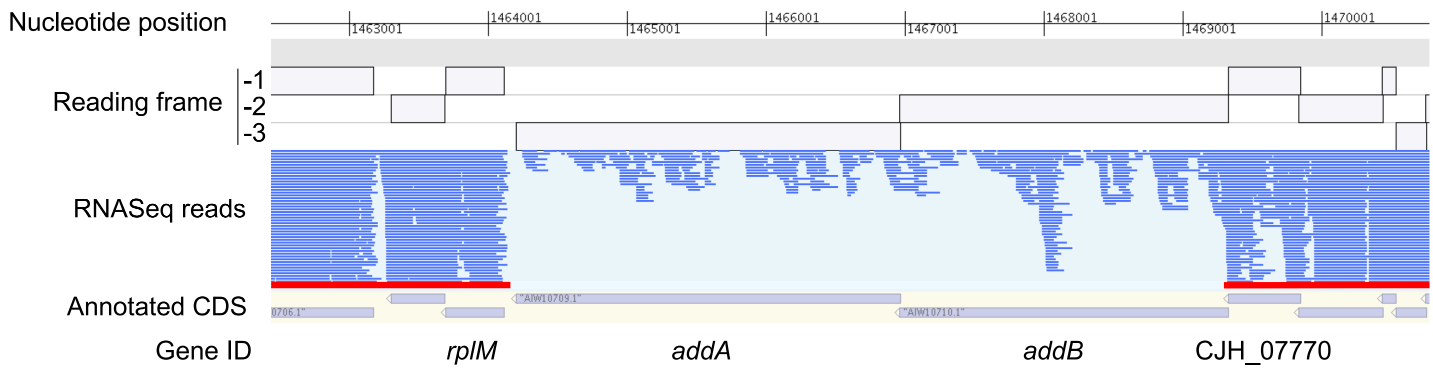

Supplementary Figure 3. Original image of Figure 2, without contrast enhancement. *C. jejuni* strain F38011 was grown for 20 hours in MH broth with different concentrations of deoxycholate. Samples were collected and subjected to pulsed-field gel electrophoresis to evaluate DNA integrity. Intact DNA remains in the well, while fragmented DNA migrates farther in the gel. Limited to no damage was observed at a concentration less than or equal to 0.05% DOC, while damage was apparent in 0.1% DOC.

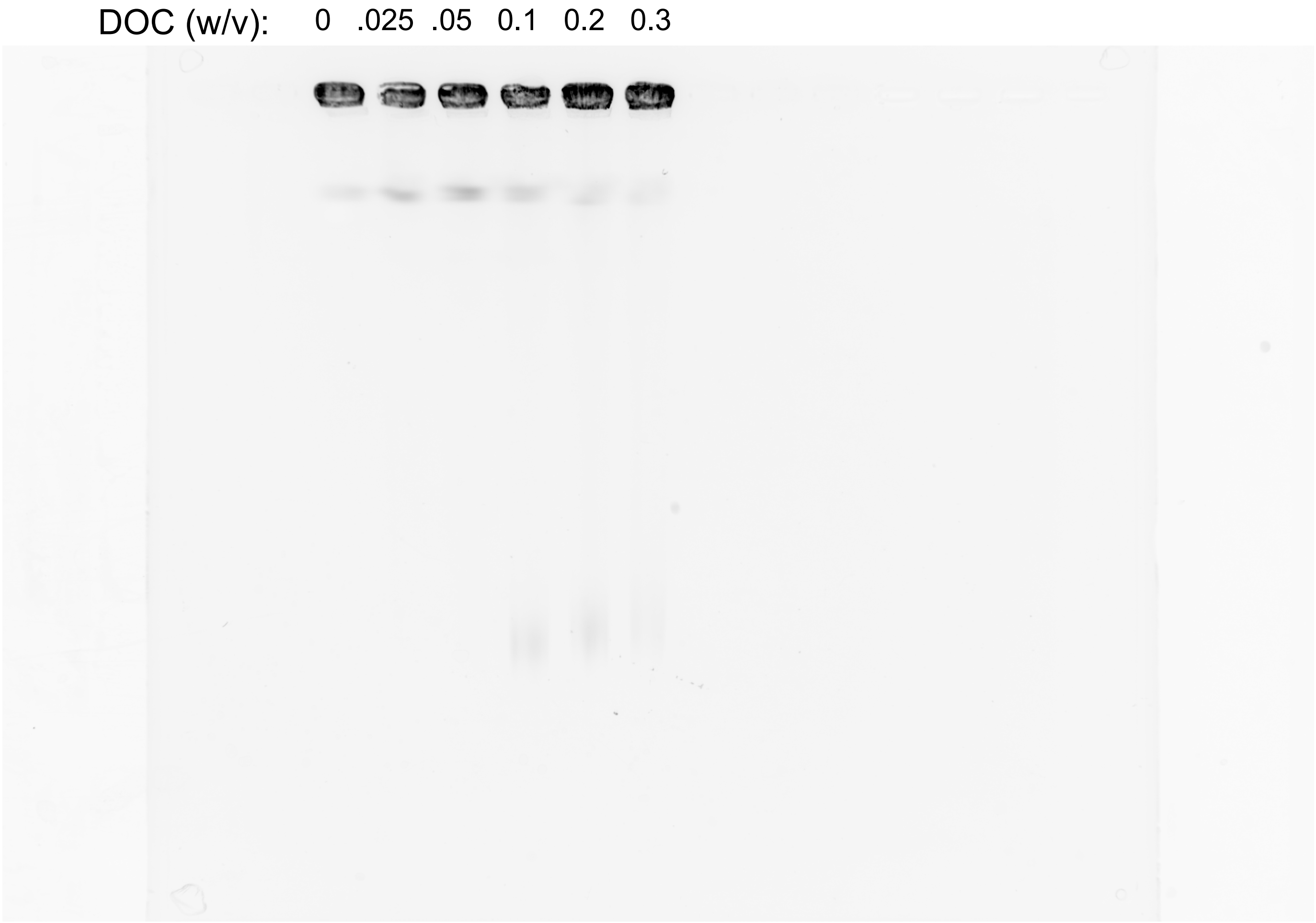

SUPPLEMENTARY TABLES

Supplementary Table 1. A detailed comparison of the putative *addA* and *addB* genes (A and B), nucleotide sequence (C and D), and gene products (E and F) from *C. jejuni* strains F38011, 81-176, and NCTC 11168.

A) Nucleotide and amino acid analysis of the putative *addA* gene

***C. jejuni* strain and gene designation**	Nucleotides	Amino acids	Calculated molecular mass (in Dalton (Da))
F38011<br>CJH_07760	2766	921	108049.67
81-176<br>CJJ81176_RS07100	2766	921	108064.73
NCTC 11168<br>Cj1481c	2766	921	108143.79

B) Nucleotide and amino acid analysis of the putative *addB* gene

***C. jejuni* strain and gene designation**	Nucleotides	Amino acids	Calculated molecular mass (in Dalton (Da))
F38011<br>CJH_07765	2367	788	93662.42
81-176<br>CJJ81176_RS07105	2367	788	93550.24
NCTC 11168<br>Cj1482c	2367	788	93641.43

C) Percent nucleotide identity of the putative *addA* gene

***C. jejuni* strain and gene designation**	F38011<br>CJH_07760	81-176<br>CJJ81176_RS07100	NCTC 11168<br>Cj1481c
F38011<br>CJH_07760	100%	98%	99%
81-176<br>CJJ81176_RS07100	98%	100%	98%
NCTC 11168<br>Cj1481c	99%	98%	100%

D) Percent nucleotide identity of the putative *addB* gene

***C. jejuni* strain and gene designation**	F38011<br>CJH_07765	81-176<br>CJJ81176_RS07105	NCTC 11168<br>Cj1482c
F38011<br>CJH_07765	100%	99%	99%
81-176<br>CJJ81176_RS07105	99%	100%	99%
NCTC 11168<br>Cj1482c	99%	99%	100%

E) Percent amino acid identity and similarity of the putative AddA protein

***C. jejuni* strain and gene designation**	F38011<br>CJH_07760	81-176<br>CJJ81176_RS07100	NCTC 11168<br>Cj1481c
F38011<br>CJH_07760	100% / 100%	98% / 98%	99% / 99%
81-176<br>CJJ81176_RS07100	98% / 98%	100% / 100%	98% / 99%
NCTC 11168<br>Cj1481c	99% / 99%	98% / 99%	100% / 100%

F) Percent amino acid identity and similarity of the putative AddB protein

***C. jejuni* strain and gene designation**	F38011<br>CJH_07765	81-176<br>CJJ81176_RS07105	NCTC 11168<br>Cj1482c
F38011<br>CJH_07765	100% / 100%	99% / 99%	99% / 99%
81-176<br>CJJ81176_RS07105	99% / 99%	100% / 100%	98% / 99%
NCTC 11168<br>Cj1482c	99% / 99%	99% / 99%	100% / 100%
